# A Cadaveric Case Report of Retroaortic Left Renal Vein in an Elderly Male With Multiple Known Comorbidities

**DOI:** 10.7759/cureus.67296

**Published:** 2024-08-20

**Authors:** Armand Cox, Cameron Kelly, Hayden Latour Callery, Whitney Main Allen, Jacob Moore, Jessica O'Bryan, Matthew D Overturf, Uzochukwu Adabanya

**Affiliations:** 1 Medicine, Edward Via College of Osteopathic Medicine, Monroe, USA; 2 Anatomical Sciences, Edward Via College of Osteopathic Medicine, Monroe, USA

**Keywords:** retroaortic left renal vein, renal anatomy, cadaveric case report, anatomical variation, nutcracker syndrome (ncs)

## Abstract

This case report describes the discovery of a type 1 retroaortic left renal vein (RLRV) in an 83-year-old male cadaveric donor with multiple comorbidities. RLRV is an anatomical variant with an estimated incidence of 0.5-3.6%, with type 1 RLRV being the most common subtype. RLRV is typically asymptomatic, which aligns with the benign nature of the anatomical variation seen in this case. However, it is important to recognize that RLRV can be symptomatic. The hallmark manifestations of renal vein entrapment, colloquially known as the "nutcracker syndrome," include hematuria, proteinuria, flank pain, and varicocele, which are concurrent with the encasement of the renal vein between the aorta and surrounding anatomical structures.

RLRV is typically diagnosed using multidetector CT (MDCT) or Doppler ultrasound (DUS). The therapeutic approach to symptomatic RLRV primarily encompasses conservative strategies, such as the administration of angiotensin-converting enzyme (ACE) inhibitors and aspirin, whereas surgical interventions are generally reserved for refractory cases, i.e., when conservative measures fail to alleviate the symptoms.

## Introduction

Retroaortic left renal vein (RLRV) is a rare anatomical variation observed during abdominopelvic dissections [1]. Typically, the left renal vein originates from the left renal hilum, and traverses anterior and posterior to the descending aorta and superior mesenteric artery respectively, before merging with the inferior vena cava (IVC). In RLRV, the left renal vein courses behind the aorta, making it susceptible to compression between the aorta and adjacent structures such as the lumbar vertebrae [1]. Anatomical variations in the course and termination of the left renal vein are categorized into four types, with varying incidences and clinical implications [2].

RLRV is typically benign and asymptomatic, but it can occasionally present with symptoms, often referred to as "nutcracker syndrome," due to the compression of the left renal vein between the aorta and other structures. Symptomatic patients may experience microhematuria, flank pain, and varicocele [1]; comorbid abdominopelvic conditions that raise intraabdominal pressures tend to increase the possibility of presenting symptoms. This report describes a case of RLRV found during the dissection of an elderly male cadaveric donor with multiple reported comorbidities.

## Case presentation

**Figure 1:**
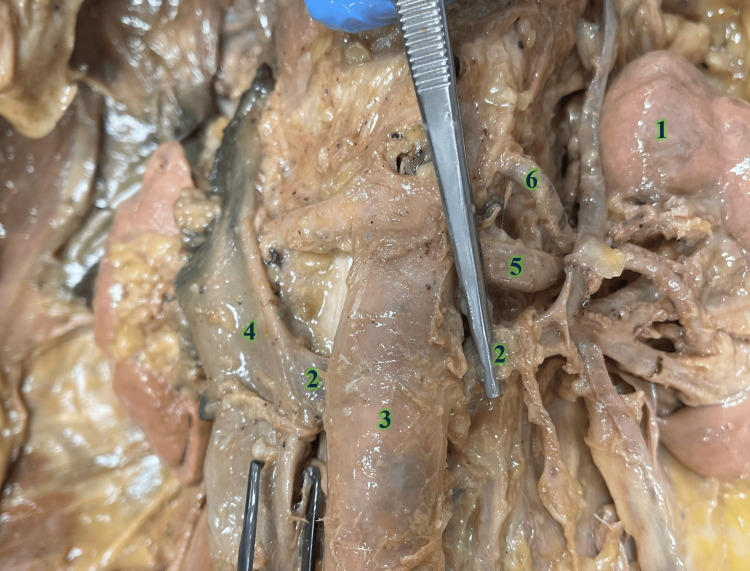
Gross imaging of the retroperitoneal space The left renal vein (2) transverses from the left kidney (1) posterior to the abdominal aorta (3) and ultimately drains into the inferior vena cava (4). Also noted are the left renal artery (5) and a left accessory renal artery (6)

During the routine dissection of an 83-year-old male cadaver, a type 1 RLRV was observed (Figure 1). Bilateral renal atrophy was also seen. The left kidney was noted to have an accessory renal artery supplying the inferior pole, although the renal capsules, parenchyma, and pelvises appeared grossly normal. The ureters followed a typical trajectory from the ureteropelvic to the ureterovesical junctions, and the bladder was normal in size and morphology. There was an absence of abdominal or inguinal hernias. The genital examination showed an anatomically standard circumcised penis with no discernible varicocele or other testicular anomalies.

Further systemic examination revealed several other pathological findings. The patient displayed diffuse muscle wasting and a cachectic physique, aligning with the documented history of adult failure to thrive. Dissection of the head, eyes, ears, nose, and throat (HEENT) showed a mildly asymmetric cranium, intact structures of the forebrain, midbrain, and hindbrain, an artificial lens implant in the right eye, normal ears and nasal passages, and a complete absence of teeth. Cardiovascular assessment was consistent with the patient’s history of arrhythmia, coronary artery disease (CAD), and congestive heart failure (CHF); findings included a left-sided pacemaker, multiple coronary bypass grafts, and sclerotic plaques in the bilateral carotid arteries. The respiratory system examination showed thickening of the parietal pleura and endothoracic fascia with predominantly normal pulmonary parenchyma, except for a small lesion in the base of the left lower lobe containing caseous debris. The gastrointestinal system examination revealed calcified lesions on the splenic capsule. This report primarily addresses the anatomical discovery of a type I RLRV in the cadaver examined.

The "cause of death" listed for this individual encompassed a comprehensive array of comorbidities, including terminal arrhythmia, CHF, stage 3 chronic kidney disease (CKD), arteriosclerosis, CAD, adult failure to thrive, dementia, and chronic obstructive pulmonary disease (COPD).

## Discussion

Cadaveric dissection plays a crucial role in medical education, enhancing understanding of anatomical relationships and variations. RLRV is a benign anatomic variation that only rarely presents with symptoms; in a retrospective study of 7,929 patient records, 0.77% of patients had RLRV, and only 6.6% of those patients were symptomatic [3]. Symptomatic patients are usually said to have the “nutcracker syndrome” or “nutcracker phenomenon”, referring to the compression of the left renal vein between the aorta and other surrounding structures (i.e., superior mesenteric artery or lumbar vertebrae) leading to a nutcracker-like shape on cross-sectional imaging. Nutcracker syndrome can fall into one of two categories, depending on the structures involved in the entrapment: anterior nutcracker syndrome is the compression of the LRV between the aorta and superior mesenteric artery, whereas posterior nutcracker syndrome occurs between the vertebral column and the aorta.

RLRV is typically associated with the posterior presentation, which is also more commonly reported. Symptomatic patients characteristically present with microhematuria, flank pain, and varicocele [1]. Patients with posterior nutcracker syndrome also have associated pelvic congestion [4]. Anatomically, the left gonadal vein typically empties into the left renal vein, whereas the right gonadal vein directly enters the IVC. In male patients, impaired venous drainage from the left gonadal (testicular) vein precipitates the development of the varicocele routinely seen. Although symptomatic cases are infrequent, pathologies (hypertension, atherosclerosis, etc.) of the aorta, and other conditions that may raise intraabdominal pressures (e.g., ascites) exacerbate this phenomenon. Compression of the left renal vein can eventually lead to elevated renal vascular resistance, consequently increasing the glomerular filtration rates of proteins and potentially leading to the rupture of delicate renal vascular structures and exudation of blood into the urine, manifesting as hematuria [1].

A detailed typology of RLRVs, including incidences and morphological characteristics has been delineated in the literature and serves to provide valuable insights into clinical significance and potential implications that are crucial for accurate diagnosis and appropriate management of patients. Type I RLRV typically joins the IVC at the conventional anatomical position (L1/L2) and has a prevalence of 0.3-1.9%. Type II RLRV connects to the IVC at a lower vertebral level (L4-L5) and may occasionally converge with the ascending lumbar veins; this morphological variant is noted in 0.4-0.9% of cases. Type III RLRV, characterized by a “venous collar” encircling the aorta, is identified in 1.5-8.7% of the population. Conversely, type IV RLRV extends caudally, posterior to the aorta, terminating in the left common iliac vein rather than the IVC, with a reported incidence of 0.16% [2]. The incidental detection of RLRV in cadaveric studies is common, reflecting its generally asymptomatic nature, as reported in various studies [1,5-6]. RLRV has also been systematically documented in cadaveric studies, further contributing to our understanding of its anatomical variations [7-9].

This case was exceptional as it included a detailed medical history resembling an inpatient progress note, providing context to the anatomical findings. In this dissection, we identified a classical type 1 RLRV, contributing to the body of evidence surrounding RLRV anatomical variations. Existing clinical and cadaveric reports document the trajectory of type 1 RLRV across genders [7,10-11]. Notably, most cases featured accompanying accessory left renal arteries, highlighting a potential anatomical association between type 1 RLRV and additional renal arteries. A separate cadaveric report on type 2 RLRV showed typical renal arterial anatomy, suggesting subtype-specific anatomical correlations, though conclusions are constrained by small sample sizes [8].

This study’s findings mirror the literature, revealing a type 1 RLRV alongside an accessory left renal artery, suggesting a recurring anatomical pattern linked to RLRV, particularly type 1. Despite these anatomical nuances, the clinical approach to RLRV remains consistent across all subtypes. Diagnosis typically involves multidetector CT (MDCT) or Doppler ultrasound (DUS), and asymptomatic RLRV generally requires no intervention [12]. For symptomatic cases, such as those presenting with posterior nutcracker syndrome, conservative management including angiotensin-converting enzyme (ACE) inhibitors and aspirin is standard, with surgical options available for cases unresponsive to medical treatment, showing high success rates [12].

## Conclusions

RLRV is a rare anatomical variant primarily identified during cadaveric dissection and less commonly noted in clinical practice, usually as an incidental finding. Symptomatic presentations, such as nutcracker syndrome, arise from the compression of the left renal vein by adjacent structures. Recognizing this variant is crucial for clinicians before conducting retroabdominal or vascular procedures, particularly in patients with recurrent symptoms.

This case underscores the need for further research into vascular anatomical variations like RLRV, to enhance renal and vascular healthcare standards. Future studies should focus on determining the prevalence and associations of RLRV in various patient cohorts to elucidate both its asymptomatic and symptomatic occurrences. Advancements in radiological imaging could refine our understanding of RLRV, potentially decreasing complications in retroabdominal and renal vascular surgeries. This report not only holds significance for clinical applications but also enriches educational content for medical students, emphasizing the need for ongoing exploration of anatomical variations to improve medical practice and education.
